# Evidence of Flicker-Induced Functional Hyperaemia in the Smallest Vessels of the Human Retinal Blood Supply

**DOI:** 10.1371/journal.pone.0162621

**Published:** 2016-09-12

**Authors:** Angelina Duan, Phillip A. Bedggood, Bang V. Bui, Andrew B. Metha

**Affiliations:** Department of Optometry and Vision Sciences, The University of Melbourne, Parkville, Australia; University of Waterloo, CANADA

## Abstract

Regional changes in blood flow are initiated within neural tissue to help fuel local differences in neural activity. Classically, this response was thought to arise only in larger arterioles and venules. However, recently, it has been proposed that a) the smallest vessels of the circulation make a comparable contribution, and b) the response should be localised intermittently along such vessels, due to the known distribution of contractile mural cells. To assess these hypotheses in human neural tissue *in vivo*, we imaged the retinal microvasculature (diameters 3–28 μm) non-invasively, using adaptive optics, before and after delivery of focal (360 μm) patches of flickering visible light. Our results demonstrated a definite average response in 35% of all vessel segments analysed. In these responding vessels, the magnitude of proportional dilation (mean ± SEM for pre-capillary arterioles 13 ± 5%, capillaries 31 ± 8%, and post-capillary venules 10 ± 3%) is generally far greater than the magnitudes we and others have measured in the larger retinal vessels, supporting proposition a) above. The dilations observed in venules were unexpected based on previous animal work, and may be attributed either to differences in stimulus or species. Response heterogeneity across the network was high; responses were also heterogeneous along individual vessels (45% of vessel segments showed demonstrable locality in their response). These observations support proposition b) above. We also observed a definite average constriction across 7% of vessel segments (mean ± SEM constriction for capillaries -16 ± 3.2%, and post-capillary venules -18 ± 12%), which paints a picture of dynamic redistribution of flow throughout the smallest vessel networks in the retina in response to local, stimulus-driven metabolic demand.

## Introduction

Blood flow and neural tissue activity need to be tightly coupled for normal neuronal function. Firstly, since the high metabolic cost of neuronal signalling dictates that only a small percentage of neurons can be concurrently active [1], the distribution of spiking cells, and therefore the energy requirements of neural tissue, is never uniform. Secondly, despite their high energy requirements, neurons are unable to store energy to the same extent as other cells [2]. The resulting regional and dynamic blood flow regulation that must follow neural activity is known as functional hyperaemia [3], and is the basis behind the blood-oxygen-level-dependent magnetic resonance signal measured in functional magnetic resonance imaging [4].

The functional hyperaemia response may be important in the development of several disease processes. For instance, in the human retina it has been shown that the change in vessel diameter following full-field stimulation with flickering light becomes reduced prior to clinically detectable retinal damage in diabetes [5,6], systemic hypertension [7] and glaucoma [8]. These studies, along with others conducted to characterise functional hyperaemia in healthy human retinal vasculature [9–11], are limited to measuring diameter changes in vessels with diameters greater than 90 μm due to insufficient contrast and resolution with conventional imaging methods [12,13].

However, more recent animal studies suggest that functional hyperaemia is initiated in much smaller vessels (3–9 μm diameter) [14] with *in vivo* studies demonstrating the ability of these small vessels to specifically and locally alter blood flow to different regions of neural tissue following stimulation [15–17] and *ex vivo* preparations beginning to determine which cells and signalling pathways are involved [14,17,18].

Therefore, the capacity to characterise this response in the smallest terminal vessels of human circulation may add to our understanding of the neurovascular response thus laying the groundwork for studying diseases. From histology, it is known that the same contractile perivascular mural cells thought to facilitate this response in animals are found in humans on vessels with diameters ranging from 5–16 μm [17], which leads to the question of whether such vessels display a measurable response to localised flicker stimulation in the human retina.

Despite an extensive body of work, the exact mechanisms behind functional hyperaemia are still unclear [19]. Recent animal studies attribute the finely tuned flow control to discrete contractile perivascular mural cells, but the literature is divided between whether pericytes [14,18] or microvascular smooth muscle cells [16,17,20] drive the change in vessel diameter to control blood flow. Regardless of this distinction, marked response heterogeneity is expected both along and between the smallest vessels, based on findings from animal research and on what is known about the distribution and morphology of the perivascular mural cells at this level of the circulation [14,16,17].

The degree of change in vessel width, if any, and the heterogeneity of the vessel response have not yet been established in human neural tissue for vessels with diameters less than 90 μm [13]. Here, we imaged the response to visible flickering light in retinal vessels less than 28 μm in diameter, and analysed the entire vessel segment to return both an average measure of diameter and a local measure of diameter at each point along the segment. Using this method, we demonstrate the ability to quantify functional hyperaemia in the smallest vessels of the retinal circulation in healthy human participants. More importantly, by retaining spatial information of vessel responses, we show heterogeneity in vessel responses following neural stimulation which does not reflect the uniform dilations seen in larger vessels with continuous smooth muscle coats.

## Materials and Methods

### Subjects

Three healthy males (aged 29, 31 and 48) with mean spherical error < 2 D, astigmatism < 1 DC, optically clear media and good fixation skills participated. Participants had no systemic disease, no ocular disease or past history of ocular surgery, no current medications, no history of smoking, no migraines, normal BMI, normal cardiovascular status and no family history of cardiovascular disease. Approval was obtained from the Human Research Ethics Committee of the University of Melbourne, and each participant provided written informed consent prior to participation. All procedures conformed to the tenets of the Declaration of Helsinki.

Participants were asked to refrain from caffeine [21] or strenuous exercise 12 hours prior to data collection. The left eye of all subjects was used for imaging. Pupils were dilated 20 minutes prior to experimentation with one drop of 0.5% tropicamide (Alcon, USA) with additional top-ups as necessary.

### Adaptive optics

Images were acquired with a flood-illumination adaptive optics ophthalmoscope described in detail previously [22]. In brief, light from an 835-nm superluminescent diode is focussed onto the centre of the region of interest and re-imaged by the pupil-conjugate lenslets of a Shack-Hartmann wavefront sensor (f = 24 mm, pitch = 0.4 mm). The measured ocular wavefront aberration is corrected using a 97-channel deformable mirror (Alpao, Montbonnot St. Martin, France), driven in real-time (20 fps) by custom Matlab software (Mathworks, Natick, MA). When root mean square (RMS) wavefront error decreased below 0.06 μm over a 7 mm pupil, a transistor-transistor logic (TTL) pulse was sent to a logic controller that also waited for a cardiac synchronisation trigger as described below. Once both signals were raised, the logic controller triggered the imaging camera (Neo sCMOS; Andor Technology PLC, Belfast, UK), operating at 200 fps. The imaging camera outputs a TTL pulse at the beginning of each 2.5 ms frame exposure, which was used to drive the imaging source [8 W supercontinuum laser filtered to produce 0.33 mW of light at 593 ± 25 nm full-width at half maximum (Fianium Ltd., Southampton, UK)]. Light was passed through 32 m of 0.37 NA, 200 μm core diameter, step-index optical fibre (Thorlabs, Newton, NJ) to reduce coherence and associated image speckle. For each acquisition series, 80 frames were collected (400 ms total acquisition).

### Imaging region

**Fig 1:**
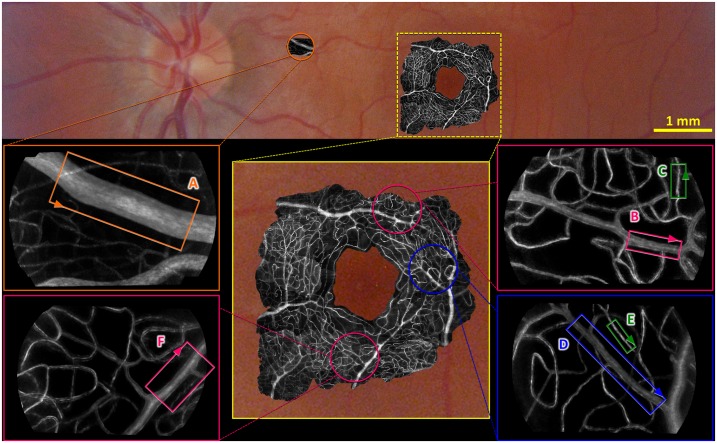
Standard retinal image (colour) of the inner retinal vasculature from one participant overlaid with motion contrast adaptive optics images (black and white) demonstrating the size and location of vessels analysed. Insets show examples of selected regions of interest. Within the insets, examples of vessel segments used for analysis have been indicated using rectangles labelled (A-F). Orange denotes a segment taken from a larger arteriole, pink from pre-capillary arterioles, green from capillaries and blue from a post-capillary venule. Arrowheads on the rectangles labelled A-F indicate the direction of flow and orientation of the corresponding segments A-F in subsequent figures.

Small regions of the inner retinal perifoveal microvascular network were selected for high-resolution imaging (Fig 1). The participant’s gaze was directed with a labelled grid to bring regions of interest into the field of view. The participant was then asked to fixate on this grid throughout the imaging sequences to maintain their eye position. The fixation grid was 5x5° in extent and printed with black ink on white paper, with grid markings spaced 1° apart. The grid was dimly back-illuminated with a white LED light source passed through a diffuser, achieving an effective luminance of 1.75 cd/m^2^ as viewed through the system with the room lights off. Nine regions of interest located 2–2.6° (580–750 μm) radially from the foveal centre were studied in total across 3 participants, from which 67 segments were selected for detailed analysis. This general eccentricity was chosen for maximal image quality. The illuminated region was 1.25° (360 μm) in diameter and included either a “major” arteriole or venule with its associated branches (8–28 μm), and the accompanying capillary bed (3–8 μm; Fig 1).

### Flicker protocol

All imaging sequences were collected with the room lights off. To establish baseline data, subjects were asked to fixate on the static, dimly illuminated grid for 20 seconds before images were taken. For stimulation, subjects fixated on the same grid location for 20 seconds whilst the imaging light (bandwidth 593 ± 25nm, full width at half maximum) was flickered on and off at 10 Hz on low power (50% duty cycle with 100% contrast, square wave, 4.7 μW during on phase, 1.25° field of view). When the flicker period ended, imaging sequences were programmed to begin at a fixed time after the latest systolic peak as described below. Five sets of baseline and 5 sets of post-flicker data were collected in alternating sequence, with 3 minutes allowed between each acquisition.

Results obtained using *in vivo* imaging techniques are heavily impacted by image quality. Factors including the participant’s tear film, fixation instability, precise alignment and the clarity of their ocular media can contribute to blur in the images we collected. This interleaved design of data collection where baseline and post-flicker data were collected in an alternating manner ensured that any noise due to the above factors should affect both conditions equally.

In a supplementary experiment, we stimulated a downstream arteriole (major vessel in field = 19.3 μm) and imaged the response to that stimulation in a corresponding upstream arteriole 7.5° (2.2 mm) away, which was 55.7 μm diameter. This was achieved by having the subject re-fixate immediately following cessation of the flicker stimulus, before acquiring the image (delay < 1.0 s, which is no greater than that inherent to cardiac cycle synchronisation explained below).

### Baseline vessel diameter variability

**Fig 2:**
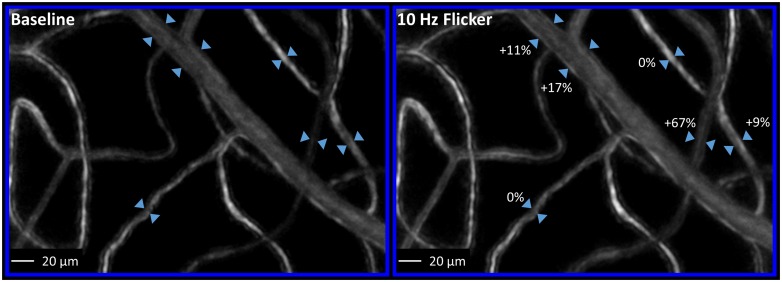
Example of a region of interest (from participant shown in Fig 1) following baseline and flicker conditions. Each region of interest was selected to contain a ‘major’ vessel and associated capillary branches. This particular region contains a venule. Note the stability in depth of focus across both conditions. To facilitate visualization of changes between pre- and post-flicker conditions for this region, see supplementary material S1 Video.

Variations in vessel diameter are known to occur over time irrespective of retinal stimulation. For instance, vessel diameter is known to change spontaneously due to vasomotion [23,24]. Diameter changes also occur with fluctuations in blood pressure [25]. The conditions of baseline and flicker stimulation were alternated during data collection to reduce the impact of these factors on our results. Specifically, five images were taken during baseline, interleaved with five images taken post-flicker. Then, all five baseline images were averaged to create a single representation for baseline, and all five post-flicker images were averaged to create a single representation for flicker stimulation (Fig 2). In addition to this, the baseline data for each region of interest was always collected on the same day as the flicker stimulation data.

### Cardiac cycle synchronisation

Over our 400 ms acquisition epoch, vessel diameter may also be affected by the cardiac cycle [26]. To mitigate this, imaging was synchronised to the participant’s pulse, monitored using an analogue optical finger pulse monitor (Model #1260, Sunrom technologies, Gujarat, India). For all conditions, the camera was programmed to begin its sequence of exposures with a time delay from the last systolic peak equal to 67% of the participant’s previous inter-beat interval, ensuring the data was collected during near-identical phases of the cardiac cycle. Image timing variations were typically accurate to within 10% of this goal; if the error was greater than this, the result was discarded and the image acquisition run was repeated.

### Image processing

Each of the five images collected for baseline underwent background subtraction and flat-fielding before they were co-registered and analysed to produce a single averaged (bright field) image and a single motion-contrast image [27,28]. The five images collected for the flicker condition were processed in the same manner. It is worth noting that intensity changes are expected in each kind of image between baseline and stimulus conditions due to changes in flow, and for this reason we focused on changes in diameter measured in each condition independently.

### Image analysis

The baseline-stimulus pair image identification key for each region was hidden with a randomly assigned number. An investigator blinded in this way to the conditions was assigned a randomised image for each region of interest and asked to select vessel segments for analysis. All vessel segments within the field that were deemed to be of sufficient image quality were selected for analysis. Selected vessel centrelines were traced manually from one region of interest in Photoshop CS6 (Adobe, USA). For each region of interest, the vessel trace was then applied to both conditions and the pair of “SEM images” detailed below. Where possible, segments were selected from the motion contrast images. Several of the smaller vessels in each field had relatively poor motion contrast due to proximity of the plane of focus [28], but acceptable bright-field contrast. In these cases, the bright-field image was analysed instead.

**Fig 3:**
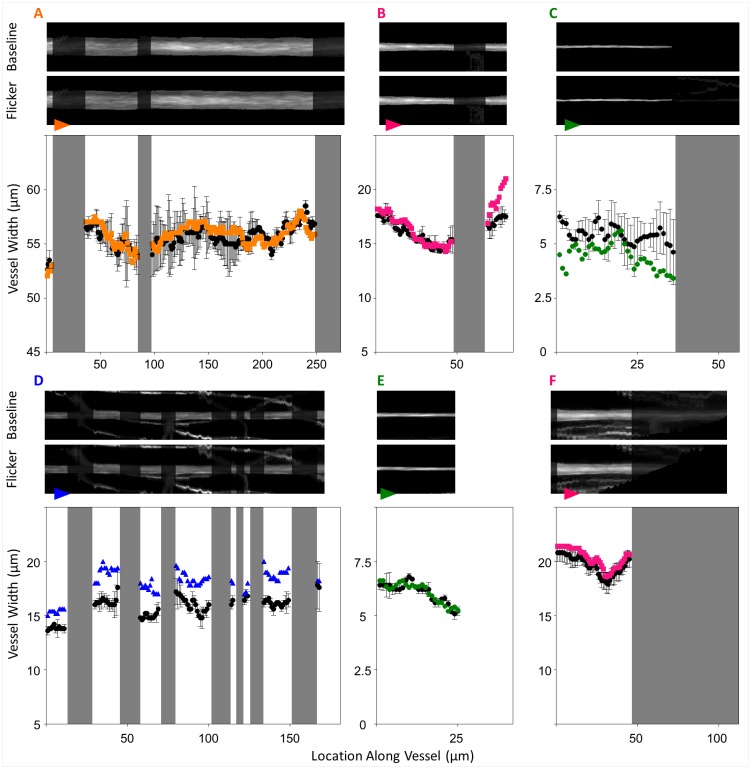
Analysis for segments highlighted in Fig 1(A)–1(F). Straightened profiles of manually traced vessels were obtained from the images (aspect ratios altered for display). During the straightening process, any visible neighbouring vessels or branches with a different orientation or curvature with respect to the vessel of interest will take on a distorted shape, causing the appearance of wavy lines seen in (D) and (F). Segments have been reoriented from Fig 1; arrowheads on each segment in this figure denote direction of flow and match the arrowheads in Fig 1. Greyed out regions indicate areas excluded from analysis due to influences of branch or crossing points, or regions of poor image quality. For the plots accompanying segments A-F, black data points denote the baseline vessel diameter and colour denotes post-stimulus diameter (orange for larger arteriole, pink for pre-capillary arteriole, green for capillary and blue for post-capillary venule). Segments B-F are examples where vessel diameter (y-axis) post-flicker was not uniform along the segment length (x-axis) as determined by the “uniformity index” described in text. When considering the average overall response, the segment taken from a larger upstream arteriole (A), capillary (E) and downstream arteriole (F) showed no change following flicker; overall constriction can be seen in a capillary segment (C); overall dilation can be seen in an arteriole (B) and a venule (D).

The vessel centreline traces were used to construct a straightened and centred representation of vessel segments against their background (see segments A–F from Fig 3). This process produces a single, centred and straight segment of interest but distorts any nearby vessels (e.g. wavy lines seen in segments D and F from Fig 3). These artefacts occur due to the varying curvatures and orientations of surrounding vessels with respect to the segment of interest, but do not affect diameter measurement of the vessel of interest.

### Edge detection

Standard vessel diameter measurement approaches fit a Gaussian model to pixel intensity data and use the full-width at half-maximum to determine vessel width [29]. However, the cross-sectional intensity profile of many of these smaller vessels did not resemble the well-behaved Gaussian shape observed in conventional imaging of large vessels, requiring development of a custom algorithm written in Matlab to robustly quantify the diameter at each point along vessel segments. Straightened segment profiles were aligned horizontally in an image array, and were subjected to edge detection analysis as follows:

1. Sub-regions with low signal-to-noise ratio, or that contained branches or crossings, were manually masked out and excluded from further analysis.
2. For each remaining pixel column, an initial edge estimate is defined by locating the adjacent pixel pair with the greatest proportional change in intensity.
3. To counteract cases of apparent non-anatomical vessel indentation that are due to noise, the following constraints were applied to the edge estimate (in order):
  - If the vessel edge deviated from horizontal by 45° or more, the edge was replaced by an average of the two adjacent rows. This process was repeated until all columns passed.
  - The most prominent bumps on capillaries in histology are 10 μm or more in length [30,31]. A rolling average filter 10 μm in length was applied to the edge data from each vessel wall.
4. Vessel edge locations were plotted for manual assessment of accuracy. If deemed unsatisfactory for any of the baseline, stimulus or associated SEM images (defined below), the data was removed from further analysis. This occurred in 27 of 94 initial candidate segments analyzed, reflecting the noisy nature of capillary image data in many segments. A major source for poor signal to noise ratio was from changes in fixation between imaging sequences. Not all segments located near the edge of the field of view were present in all 5 imaging sequences used to create the averaged image. As the “major” arteriole or venule was always centred in the region of interest, these segments were often capillaries. Additionally, due to the nature of blood vessel distribution throughout the retina, parts of some vessels, especially capillaries, dive into and out of the plane of focus selected for imaging. This produced inconsistent focus and image quality for those segments.

With the edges located, the distance between the two edges (i.e. vessel diameter) was calculated for each column and the identity of the vessel decoded to print the results.

### Statistical analysis

Estimating uncertainty: The imaging light source for our non-invasive method of imaging has a wavelength close to the peak for haemoglobin absorption. This set up provides contrast partially derived from the differential absorption of light by haemoglobin in thin columns (pre-capillary arterioles or post-capillary venules) or single erythrocytes (capillaries), and partially from phase contrast [28]. We estimate these effects to be comparable in magnitude, as contrast is roughly doubled when changing from 670 nm light (where phase contrast dominates) to 570 nm light. Overall, contrast is low compared to some other methods since no contrast dyes are used. To improve signal to noise ratio, we averaged data across the 80 frames collected over 400 ms for each imaging sequence, creating 5 baseline images and 5 post-flicker images. We then averaged data across the 5 images to create a single image for each condition. Although signal to noise ratio is improved, averaging data collected from different time points introduces variability into the data as it is known that both image quality and baseline vessel diameter can change over time due to systemic factors. It is difficult to gauge the impact of this variability with traditional statistical means since repeating the analysis with a subset of the images would result in images with reduced signal-to-noise. To circumvent these limitations we devised the concept of “SEM images”. These images were created by first calculating and doubling the standard error of the mean intensity for each pixel across all 5 imaging sequences for a given condition, thus providing an estimate of vessel variability over time. This value is then added to or subtracted from the averaged image, to generate two images that respectively show ± 2 SEM variability. Each of the ± 2 SEM images were then analysed with the same edge detection algorithm as used for the averaged image. To be conservative, the 2 SEM image with the largest difference from the baseline diameter was used to produce the 2 SEM confidence intervals (e.g. black error bars in Fig 3A–3F).

### Uniformity index

We created a “uniformity index” to determine whether or not each vessel showed heterogeneity in response along its length. This index represents the probability that any apparent locality in the response could have been produced by random noise. In other words, the index is directly analogous to a p-value assessing the null hypothesis that the vessel showed a uniform response; significant values (p < 0.05) indicate that the response was not uniform. As with regular p-values, it is important to note that the uniformity index does not directly imply the size of the effect.

To produce the uniformity index for each vessel segment, the percentage response at each location along the segment was assigned to one of two clusters using the k-means approach [32]. This is an iterative approach in which locations are first randomly assigned to one of two clusters, and then progressively re-assigned based on whether the value at each location is more similar to the centroid of one cluster or the other until a stable solution is obtained. This process is repeated many times to minimize bias introduced by the random initial configuration. Once assigned to a cluster, the longest contiguous length of pixels that fell in the same cluster was determined. This length was compared to the probability of observing a contiguous section of the same length if the cluster assignment had instead been made at random (analogous to the likelihood of tossing *k* heads from *n* coin tosses). Since this probability describes the probability of observing our results by chance, it is directly analogous to a p-value. We chose a criterion of 0.05 as a cut-off threshold for significance.

## Results

### Baseline vessel diameter quantification

**Fig 4:**
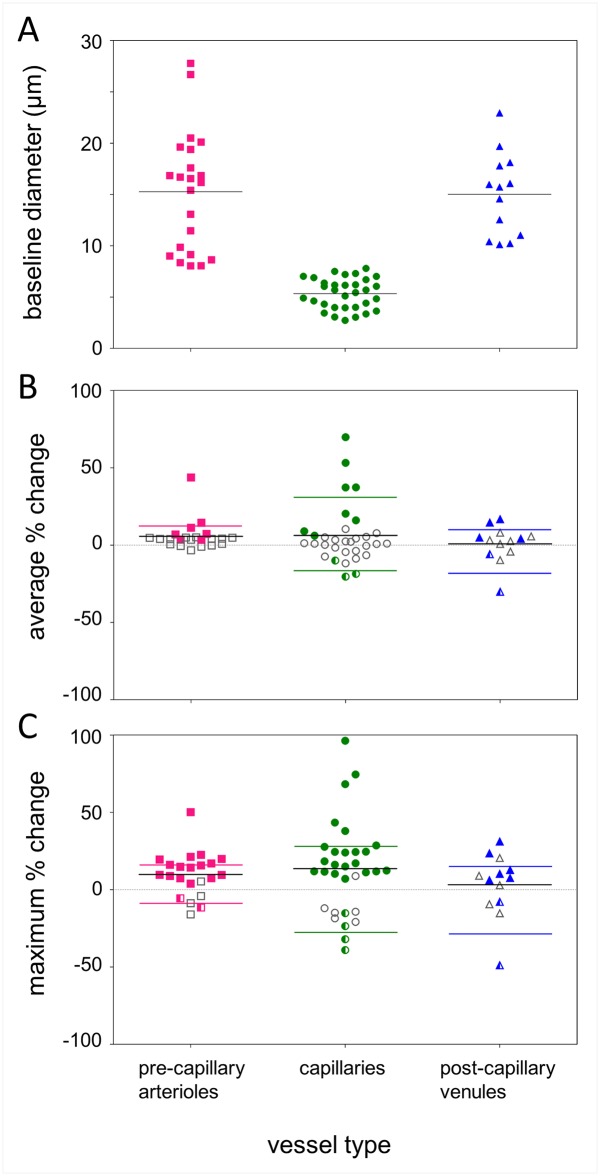
Baseline diameters and flicker-induced changes for pre-capillary arterioles, capillaries, and post-capillary venules. (A) Distribution of baseline vessel diameters (y-axis) by vessel type (x-axis) for all 67 segments included in the data set. Solid black line shows mean vessel diameter for each type of vessel. (B) Distribution of the average percentage change (y-axis) across each of the 67 segments post-flicker, grouped by vessel type. (C) Distribution of the largest regional percentage change (y-axis) seen at some point along each of the 67 segments post-flicker grouped into vessel types. For panels (B) and (C), coloured symbols show responding segments whose 2 SEM error bars did not encompass zero (see Fig 5A), i.e. segments with a definite measurable change. Filled symbols indicate a definite dilation, and half-filled symbols indicate a constriction. Unfilled grey symbols represent “non-responding” segments. These are either segments that did not show a measurable change post-flicker or whose baseline variability was greater than the change measured, which are all classified as “non-responding”. Solid black lines represent the overall average of all data points for each vessel type. Coloured lines represent the average of responses from segments with a valid response, with the average for dilations alone and constrictions alone displayed individually.

After removing low signal to noise data as explained above, a total of nine regions of interest from the inner retinal vasculature surrounding the macula of each participant were imaged and identified as containing a “major” pre-capillary arteriole or post-capillary venule based on the direction of blood flow discernible from imaging at 200 fps. From these regions, 67 segments were selected, made up of 22 pre-capillary arterioles, 32 capillaries and 13 post-capillary venules, with baseline diameter range of 3–28 μm (Fig 4A). The mean ± SD diameter for each vessel type was as follows: 15.3 ± 5.8 μm for arterioles, 5.3 ± 1.5 μm for capillaries and 15.0 ± 4.0 μm for venules (Fig 4A).

Here, we have chosen to define capillaries as vessels with diameters < 8 μm based on histological studies in human retina [33] and our own observations of single-file erythrocyte flow being always apparent in vessels of this diameter and smaller [28,34].

### Vascular response to flicker

All vessel types selected from the peri-macula region showed the ability to respond to flicker. This is illustrated in Fig 3 where segments highlighted in Fig 1 are shown before and after flicker stimulation. Here, an overall dilation can be seen in segments B and F (pre-capillary arterioles) and segment D (post-capillary venule). An overall constriction can be seen in segment C (capillary).

Vessel responses were quantified in two ways: firstly, by using the average response for each segment (Fig 4B) and, secondly, because the response was often not uniform along the segment, by using the largest regional response for each segment from any given position along the length of that segment (Fig 4C).

### Average responses

**Fig 5:**
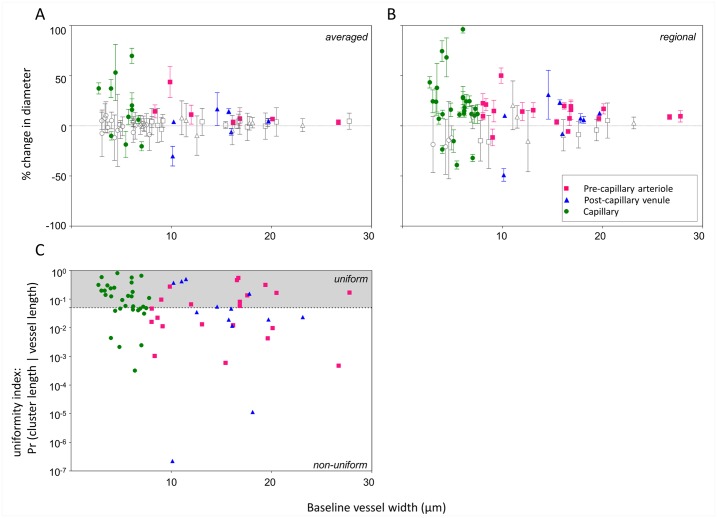
Relative responses and uniformity indices for vessels according to size. (A) Average response for each segment (B) largest regional response for each segment for all 67 segments post-flicker. Data is expressed as the percentage change from baseline diameter (y-axis) as a function of baseline vessel size (x-axis). Error bars are 2 SEM values for each segment. Coloured symbols represent segments that displayed a definite response defined as “responses” whose 95% confidence intervals do not encompass zero. Grey symbols represent segments classified as “non-responders” where the 95% confidence interval encompasses zero. (C) Uniformity index for all segments studied, i.e. the y-ordinate plots the probability of the observed pattern if apparent non-uniformities are caused by chance. Segments with data points below the dotted line (p = 0.05) are taken as demonstrating locality (non-uniformity) in their response.

Of the 67 segments studied, 35% produced a definite average response (Fig 4B) defined as segments whose ± 2 SEM error bars did not encompass zero (Fig 5A). Of the definite average responses, dilation was most common, with mean ± SEM overall percentage dilations for each vessel type as follows: 12.9 ± 5.3% in pre-capillary arterioles, 31.1 ± 7.9% in capillaries and 10.0 ± 3.3% in post-capillary venules (Fig 4B). Importantly, 7% of vessel segments showed an average constriction (Fig 4B), only seen in capillaries (-16.3 ± 3.2%) and post-capillary venules (-18.1 ± 12.2%). The larger upstream feeder arteriole (segment A, Fig 3) showed no average response.

There were some vessel segments that did not demonstrate a measurable average response (Fig 4B, unfilled grey data points). These vessels either showed zero proportional change overall, or did not demonstrate a response that fell outside of the baseline vessel diameter variability (the ± 2 SEM error bars encompassed zero in Fig 5A). When non-responders were taken into account, the mean ± SEM overall percentage dilations for each vessel type were: 5.7 ± 2.0% in pre-capillary arterioles, 6.2 ± 3.4% in capillaries and 0.8 ± 3.3% in post-capillary venules.

### Regional responses

Due to variations in the magnitude and direction of vessel diameter changes, responses often cancelled out when averaged along the entire length of a vessel segment. Thus, for each of the 67 vessel segments, we also considered the single largest definite change in diameter where the ± 2 SEM error bars did not encompass zero (Fig 5B). In this way, a definite regional response (within the 10 micron resolution imposed by the analysis method) was measured in 77% of vessel segments.

Where definite regional responses were detected, the lower bound of the 95% confidence interval was conservatively used to report magnitude (Fig 4C). Dilations were the most common regional responses (pre-capillary arterioles: 10 ± 2.5%, capillaries: 19.7 ± 4.8%, post-capillary venules: 8.9 ± 2.8%, Fig 4C). The largest regional response of two pre-capillary arterioles (-4.1 ± 0.9%), four capillaries (-21.8 ± 6.7%) and four venules (-24.2 ± 18.4%) were constrictions (Fig 4C). The largest regional response of the upstream feeder arteriole (segment A, Fig 3) was a dilation of 1.4%.

There were some vessel segments that did not demonstrate a measurable response at any point along the vessel segment (Fig 4C, unfilled grey data points). These situations arose either from segments that had no regional change in diameter between conditions, or all regions of response along the segment did not fall outside of the baseline vessel diameter variability (the ± 2 SEM error bars encompassed zero in Fig 5B). When non-responders are taken into account, the mean ± SEM for each vessel type were: 9.8 ± 3.0% in pre-capillary arterioles, 13.6 ± 5.3% in capillaries and 3.2 ± 5.8% in post-capillary venules.

### Evidence of locality in vessel response

Vessel responses were often not uniform along the length of each segment. Using the uniformity index, we show that of the 67 segments, 30 segments had statistically significant locality indices i.e. had local regions of vessel diameter modifications that were not likely due to chance (p < 0.05; Fig 5C). For more direct illustration, segments B, C, D, E and F in Fig 3 all had significant locality indices of p < 0.05.

## Discussion

Our results document how the smallest vessels of the human inner retinal circulation respond to a locally delivered flickering light stimulus. Whilst functional hyperaemia has been demonstrated in vessels of equivalent size *in vivo* in anaesthetised rat retina [16], this is the first study to demonstrate functional hyperaemia in human vessels with baseline diameter < 30 μm, and the first study to demonstrate that a very small (360 μm) flicker stimulation produces a measurable change in the coupled vessels *in vivo*.

### Dilations

The most common response was dilation, consistent with the 2–5% dilations seen in human retinal arterioles and venules (with diameters > 100 μm) following 8–12 Hz flicker [11,35,36]. This is also in line with the vasodilation (5–7%) measured *in vivo* in vessels < 40 μm in rat retina [16].

However, the magnitude of vasodilation measured in this study was much larger than in the above studies that examined larger vessels. When considering the overall response of each segment, vessels < 8 μm showed the greatest proportional change, dilating by 31% on average compared to vessels > 8 μm which dilated by 10–13% (Figs 4B & 5A). When considering regional responses within each segment, more segments were found to have a measurable response to flicker stimulation. The majority of regional responses were also dilations, with vessels < 8 μm showing regional dilations of 20% on average compared to vessels > 8 μm which had regional dilations of 9–10% (Figs 4C & 5B). Both methods produce results that are more comparable to the vasodilations (10–18%) seen *in vivo* in mouse cortex microvasculature (2–12 μm diameter) following whisker pad [14,17] and forepaw stimulation [37]. This paints a consistent picture that the smallest class of vessels are capable of larger proportional changes than larger vessels.

For Newtonian fluid flow, vessel resistance is inversely proportional to vessel radius to the fourth power, so a small change in vessel diameter should produce a large change in vessel resistance as described by Poiseuille’s Law [38]. However, whilst this relationship is thought to approximate haemodynamics in the larger vessels, it will not hold in capillaries where there is single file erythrocyte flow. In fact, the average diameter of a human erythrocyte [39] is greater than the diameter of many capillaries, resulting in the erythrocytes having to deform markedly in order to pass through [40]. Thus, these large proportional changes in capillary diameter may contribute even more significantly to blood flow and distribution than a large proportional change in pre- or post-capillary vessel diameters.

In comparison to the vasodilation seen in the larger up- or down-stream vessels, the proportional dilation shown in the smaller vessels here are much greater, lending support to the idea that vessels < 30 μm contribute sizeably to functional hyperaemia in neural vasculature [14,16,17], as greater responses are unlikely to occur passively. For instance, following whisker stimulation, vessel segments with diameters < 30 μm in mouse cortex co-localising with a smooth muscle cell dilated by 10%, but measurements made in downstream segments without smooth muscle cell co-localisation dilated by only 1% [17]. Ultimately, comparison of vessel response time course between upstream and downstream vessels is needed to determine where functional hyperaemia is initiated, but this is technically infeasible with our protocol as the intensity of the imaging light interferes with the time course of the response.

The two main dissimilarities in our protocol that may account for the differences in response magnitude compared to previous human data are the size of the flicker stimulus used (1.25° compared to 30° diameter) and the size of the vessels studied (< 30 μm compared to > 90 μm) [13]. To determine the effect of our more focal stimulus on larger vessels, we conducted a supplementary experiment where we stimulated a previously selected region of interest but measured the response in the larger upstream feeder arteriole (56 μm diameter, Fig 3A) following the downstream focal stimulation. There was no detectable overall response in the feeder arteriole whereas overall responses of dilation (5%) and constriction (-18%) of vessels downstream from this vessel were far greater, suggesting that our focal stimulus produced a region-matched response and did not have significant effect on the upstream feeder vessel some distance away.

### Constrictions

Several segments showed an overall constriction following stimulation (Fig 4B), while several more showed definite narrowing at some point along the vessel (Fig 4C). Constrictions provide strong evidence for a local and active process of flow modulation in vessels < 30 μm, as they are unlikely to occur subsequent to increased blood flow upstream.

Vasoconstriction in response to functional stimulation has not been noted in human retinal vasculature, but has been observed in vessels < 30 μm in animal studies. Examples of focal constriction were observed following electrical [18] and neurotransmitter [30] stimulation of a pericyte in isolated rat retina, following application of a prostaglandin analogue *in vivo* in rat brain [20], and following optogenetic stimulation of a smooth muscle cell *in vivo* in mouse brain [17].

Constrictions have been proposed as a means to adjust blood flow distribution towards regions of neural activation as part of an overall, and perhaps coordinated, network response [15,37,41]. For instance, with increasing distance from the centre of neural activity there is an observable increase in the magnitude of constriction responses [41]. Based on this theory, both the local nature of our stimulus and the location of imaging may have contributed to the observation of occasional constriction responses. At our imaging locations (1.4–3.2° from the foveal centre), the retinal ganglion cells supplied by the vasculature we have imaged are laterally displaced from their photoreceptors by 1.1–1.8° (311–524 μm) [42]. As our region of interest was 1.25° (360 μm) in diameter, some of the photoreceptors stimulated will have activated ganglion cells outside of our imaging region, causing a radial mismatch between stimulated neural tissue and the vasculature being imaged. However, in the 9 retinal areas that we studied across 3 subjects we found no evidence of a foveal-centred radial response pattern, suggesting that flow redistributions have higher complexity than can be explained by areal considerations alone.

### Venule response

The sizeable dilation and constriction responses of venules in this study were not anticipated from the small dilation (1%) in rat retina venules following full-field flicker stimulation [16]. Venules were also reported to rarely display significant diameter changes in mouse cortex [17].

Although further study is required to differentiate whether our measured venule responses were actively or passively generated, the instances of constriction (Fig 4B & 4C) suggest there may be contractile mural cells present on these vessels of the human retina. There may be structural differences in circulation across species that explain why this has not been reported previously. For example, the size difference between retinal arterioles and venules is greater in rats than in humans, so human venules may undergo a greater change in size to receive increased blood flow [16].

### Response heterogeneity

Vessel responses were variable along the length of most vessels analysed (Figs 3B–3F and 5C). Of the 67 segments analysed, 35% displayed a definite overall response (Fig 5A) but 77% of segments displayed a definite response (Fig 5B) at some point along their lengths. The more conservative uniformity index confirmed locality in 45% of vessel segments. Whilst the exact location of perivascular cells cannot be identified in human participants, the distribution of discrete contractile perivascular mural cells on vessels < 30 μm seen in histology [17,43] do predict that non-uniform responses should be observed along and across vessel segments.

When considering each region of interest, vessel responses were also highly variable across the vascular network (Fig 2). In addition to variations in response direction and magnitude, some vessels had no measurable response at all. In animal studies, non-responding vessel segments in the smaller vessels are attributed to a lack of perivascular mural cells in those vessels [14,17,18].

A reason for creating heterogeneity in blood flow has been put forth in a network model of blood flow regulation that suggests tissue oxygenation can be modulated by increasing or decreasing heterogeneity in erythrocyte transit times through vascular beds [44,45].

## Conclusions

Our results show a) significant dilations in the smallest vessels of the human retina following focal flicker stimulation; b) a greater proportional response in these vessels than is observable in the larger vessels up- or down-stream; c) heterogeneity in the response both along a vessel segment and throughout the vascular bed, supporting the proposed facilitation role of the intermittently distributed perivascular mural cells; d) constrictions in some vessels, supporting a role for the response in local redistribution of blood flow across the network according to metabolic need. Altogether these observations show that the smaller vessels of the human retinal vasculature contribute significantly to functional hyperaemia, and lend support to the idea that focal neural activation results in complex blood flow redistribution. Whether active flow redistribution is confined to a specific class of vessel, and the spatial extent over which flow redistribution occurs, are interesting points for further study. Finally, imaging the smallest vessels might aid in earlier disease detection given that the proportional response appears to be greater than those seen in larger vessels.

## Supporting Information

S1 Video Animation of pre- and post-flicker images from the region of interest shown in Fig 2. This region contains a venule and associated capillaries. Alternating between the registered pre- and post-flicker images aids visualization of vessel diameter changes induced by localized flicker stimulation.
(AVI)
